# Transient exposure to rotenone causes degeneration and progressive parkinsonian motor deficits, neuroinflammation, and synucleinopathy

**DOI:** 10.1038/s41531-023-00561-6

**Published:** 2023-08-11

**Authors:** Amber D. Van Laar, Katherine R. Webb, Matthew T. Keeney, Victor S. Van Laar, Alevtina Zharikov, Edward A. Burton, Teresa G. Hastings, Kelly E. Glajch, Warren D. Hirst, J. Timothy Greenamyre, Emily M. Rocha

**Affiliations:** 1 Pittsburgh Institute for Neurodegenerative Diseases, University of Pittsburgh, Pittsburgh, PA USA; 2 Department of Neurology, University of Pittsburgh, Pittsburgh, PA USA; 3 Department of Pharmacology & Chemical Biology, University of Pittsburgh, Pittsburgh, PA 15213 USA; 4 Geriatric Research, Education and Clinical Center, VA Pittsburgh Healthcare System, Pittsburgh, PA 15240 USA; 5 Neurodegenerative Diseases Research Unit, Biogen, Cambridge, MA 02142 USA

**Keywords:** Parkinson's disease, Parkinson's disease

## Abstract

Individuals with Parkinson’s disease (PD) typically receive a diagnosis once they have developed motor symptoms, at which point there is already significant loss of substantia nigra dopamine neurons, α-synuclein accumulation in surviving neurons, and neuroinflammation. Consequently, the point of clinical presentation may be too late to initiate disease-modifying therapy. In contrast to this clinical reality, animal models often involve acute neurodegeneration and potential therapies are tested concurrently or shortly after the pathogenic insult has begun rather than later when diagnostic clinical symptoms emerge. Therefore, we sought to develop a model that reflects the clinical situation more accurately. Middle-aged rats (7–9 months-old) received a single daily intraperitoneal injection of rotenone for 5 consecutive days and were observed over the next 8–9 months. Rotenone-treated rats showed transient motor slowing and postural instability during exposure but recovered within 9 days of rotenone cessation. Rats remained without behavioral deficits for 3–4 months, then developed progressive motor abnormalities over the ensuing months. As motor abnormalities began to emerge 3 months after rotenone exposure, there was significant loss of nigral dopaminergic neurons and significant microglial activation. There was delayed accumulation of α-synuclein in neurons of the substantia nigra and frontal cortex, which was maximal at 9 months post-rotenone. In summary, a brief temporally-remote exposure to rotenone causes delayed and progressive behavioral and neuropathological changes similar to Parkinson’s disease. This model mimics the human clinical situation, in which pathogenesis is well-established by the time diagnostic motor deficits appear. As such, this model may provide a more relevant experimental system in which to test disease-modifying therapeutics.

## Introduction

Parkinson’s disease (PD) patients typically come to the attention of a physician once motor symptoms have begun, and by the time of diagnosis, significant neuropathological changes have already occurred^1–3^. Early parkinsonian symptoms (resting tremor, rigidity, bradykinesia, etc.) are indicative of nigrostriatal dopamine (DA) deficiency and are a clinical manifestation of a decompensated disease state as DA levels become insufficient for normal motor control^4^. This delayed clinical presentation in relation to the onset of disease pathology suggests that relatively silent neuropathogenic processes have been underway well in advance of symptom onset and the start of treatment. As a multifactorial neurodegenerative disease, the manifestations and cadence of PD progression are impacted by genetic, epigenetic, and environmental factors, all of which are influenced by the passage of time. As a result, there is a high hurdle to overcome in the development of a disease-modifying treatment which, in turn, emphasizes the need to develop better preclinical PD animal models.

PD research has relied on a variety of model systems to understand the complexities of this slowly progressive disease. These model systems have yielded a more comprehensive understanding of the neuropathogenic mechanisms underlying the disease, leading to the development of novel therapeutic strategies currently under clinical investigation. However, the indolent time course of idiopathic PD has complicated the development of accurate predictive disease models and, thereby, the discovery of relevant therapeutic strategies. Induction of early neuropathogenic changes prior to the onset of a motor phenotype, followed by a progressive neuropathology that is coupled with the onset of symptoms has been difficult to capture in a single parkinsonian animal model. Also, attempts to translate neuroprotective therapies identified in preclinical work into clinical practice have generally failed^5,6^, which may be directly attributed, at least in part, to the models used to identify therapeutic strategies.

Toxicant-induced models of PD typically recapitulate moderately advanced stages of PD, such as the marked degeneration of the dopaminergic nigrostriatal pathway that is associated with a moderate to severe parkinsonian phenotype. Further, these behavioral and pathologic changes are often elicited within a few days, contrasting with the pattern of clinical disease progression in individuals with PD. Meanwhile, genetic models can be slow to develop, and some may not show robust pathological changes or behavioral phenotypes. Certain model systems also fail to capture the non-motor symptoms of PD or other neuropathological features like the presence of Lewy bodies, extra-nigral α-synuclein accumulation, or PD-associated neuroinflammatory changes.

When using toxicant-induced parkinsonian models for preclinical testing of therapeutic strategies, putative treatments are often started well before symptom onset – either before, or concurrently with, toxicant administration, a scenario with relatively limited clinical relevance. While these preclinical studies have yielded insights about potential therapies, the models used have exhibited a limited ability to test therapeutics when the parkinsonian phenotype is still mild yet the neuropathologic processes are already well underway. Replicating the silent, ongoing neuropathogenic processes that occur prior to reaching the threshold of clinical symptoms may be essential to more realistically test potential therapeutic strategies.

In designing an ideal model of PD, it is necessary to consider what is known about the etiology and pathogenesis of the human disease in aging adults. Epidemiologic studies have linked environmental exposure to mitochondrial toxicants with an increased risk of PD^7–10^, and such exposures may occur long before clinical presentation of the disease^11,12^. We have demonstrated that chronic exposure to the pesticide rotenone – a mitochondrial complex I inhibitor that is epidemiologically linked to PD – can cause a parkinsonism phenotype in rats^7,13,14^. However, the subacute dosing regimen and acute phenotypic onset of this model limits its utility in studying long-term disease progression or therapeutic intervention after symptoms appear.

In this context, we hypothesized that a brief, transient exposure to rotenone, would initiate a neurodegenerative cascade leading to a delayed, spontaneous and progressive parkinsonian phenotype. Here we show that after a brief (5 day) exposure of aged rats to rotenone, there is a 10-week latent period, followed by a delayed and progressive gait disturbance. At the time point when motor symptoms emerge, there is evidence of ongoing nigrostriatal degeneration and neuroinflammation, recapitulating similar motor and pathologic features observed in the human disease. Over the ensuing months, there is progressive microglial activation and delayed α-synuclein in substantia nigra and cerebral cortex. The delayed onset of motor abnormalities as well as progressive neuropathological features, provides a unique opportunity to evaluate both pathogenic mechanisms and therapeutic interventions at clinically relevant time points, and provides experimental support for the hypothesis that remote exposures can trigger progressive pathogenic cascades resulting in delayed clinical presentation in patients.

## Results

### Brief rotenone exposure triggers a delayed and progressive parkinsonian phenotype

**Fig. 1:**
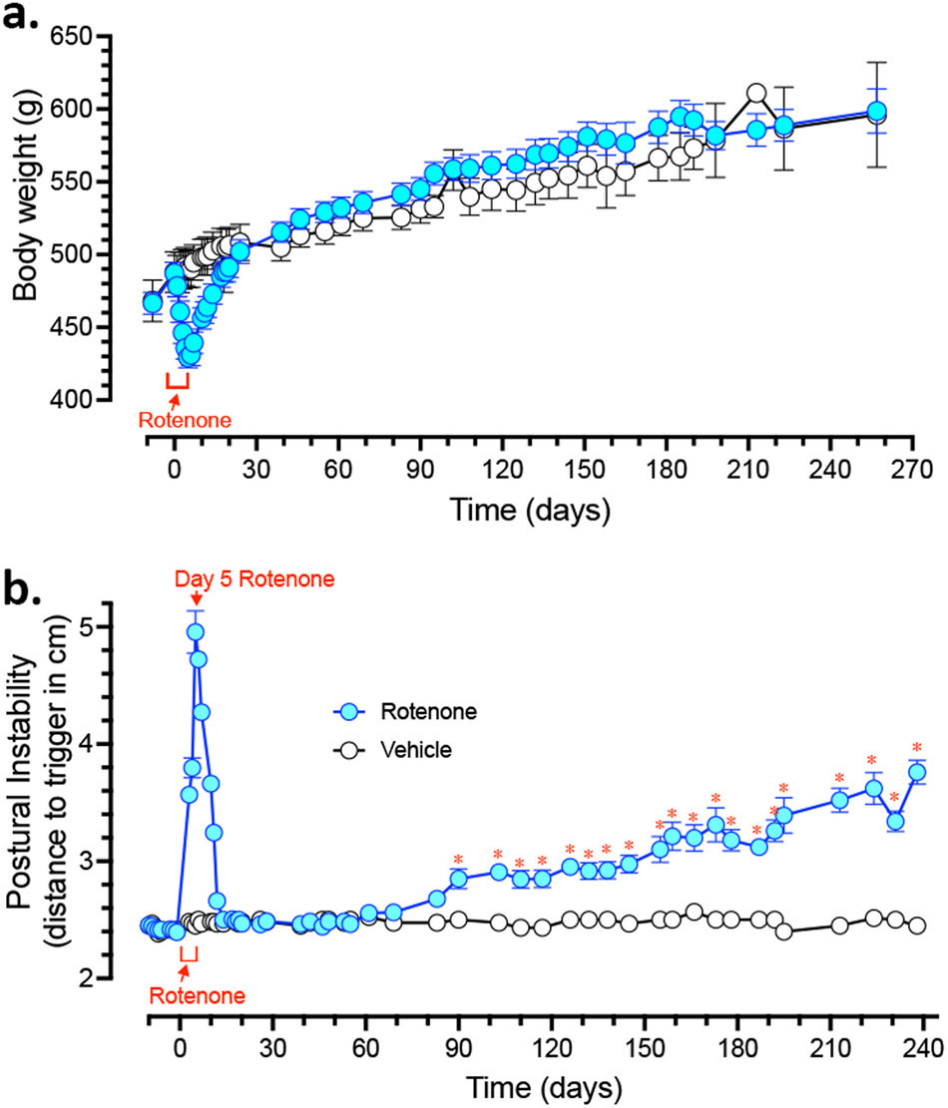
Transient rotenone exposure triggers delayed and progressive motor deficits. Male (7–9 month old) Lewis rats received a single intraperitoneal (i.p.) injection of either vehicle or rotenone (2.8 mg/kg) for 5 consecutive days. Rats were euthanized either 1-,3-,6-, or 9-months following final i.p. injection. Total body weight for vehicle treated rats (black lines) and rotenone treated rats (blue lines) were initially monitored daily and eventually weekly for the full duration of the study (**a**). All rats underwent postural instability test (PIT) training for 2 weeks prior to receiving their first administration of either vehicle or rotenone (**b**). All rats were tested initially daily and eventually weekly on PIT (distance to trigger in cm). Blue lines represent mean PIT values (cm) ±SEM of all rotenone treated animals, and Black lines represents all vehicle treated animals mean PIT values (cm) ±SEM at each corresponding time point (*p < 0.05 rotenone vs. vehicle control; *n* = 4–6 per group). Data analyzed using a repeated measures and Sidak’s multiple comparisons test.

Adult male rats (7–9 months old) were dosed with either vehicle or 2.8 mg/kg rotenone for 5 consecutive days, based on our previous observations, this dosing regimen caused an acute parkinsonian movement disorder in rats without evidence of nigrostriatal degeneration. Rats that received rotenone showed weight loss of ~12%, that was significantly different from baseline by day 3 of treatment (Fig. 1a; all times are with respect to the first day of rotenone administration). All weights returned to baseline within 3 weeks of the last rotenone treatment, and by Day 18, rats dosed with rotenone looked clinically indistinguishable from the vehicle group. Thereafter, both vehicle- and rotenone treated rats gained weight at the same rate. By study completion, all rats had gained weight, with an average increase of 22% compared to baseline (Fig. 1a).

Postural instability is a hallmark feature of PD and is a measurable parkinsonian phenotype in rodents^13^. We utilized the **P**ostural **I**nstability **T**est (PIT) to assess motor dysfunction before, during, and for months after a brief 5-day exposure to vehicle or rotenone. The PIT assesses gait disturbances by measuring the distance before a compensatory contralateral forelimb movement is triggered^13,15^. We previously showed that in rotenone-treated rats, PIT abnormalities are obviated by apomorphine and therefore reflect nigrostriatal function^13^. At baseline, rats averaged 2.5 cm of displacement before triggering a compensatory limb movement (Fig. 1b). Treatment with rotenone caused an acute gait disturbance in rats (*F*_1,22_ = 135.6; *p* < 0.0001, mixed effects repeated measures) that was significantly different from controls by Day 3 of treatment (*p* < 0.0001) and reached a maximum at Day 5 (Fig. 1b). PIT performance returned to baseline (2.5 cm) by Day 14 (9 days after discontinuing rotenone) and remained at baseline, indistinguishable from vehicle treated rats for 10 weeks. At Day 90, without further intervention, performance on the PIT began to worsen only in rats previously treated with rotenone (*p* < 0.05). PIT scores continued to worsen for this group and gradually increased over the next 6 months, reaching a maximum of 3.78 ± 0.09 cm (*p* < 0.01) at Day 238. Vehicle-treated animals remained at the initial baseline of 2.5 cm throughout the duration of the study (Fig. 1b).

### Substantia nigra dopaminergic neurodegeneration following rotenone exposure

**Fig. 2:**
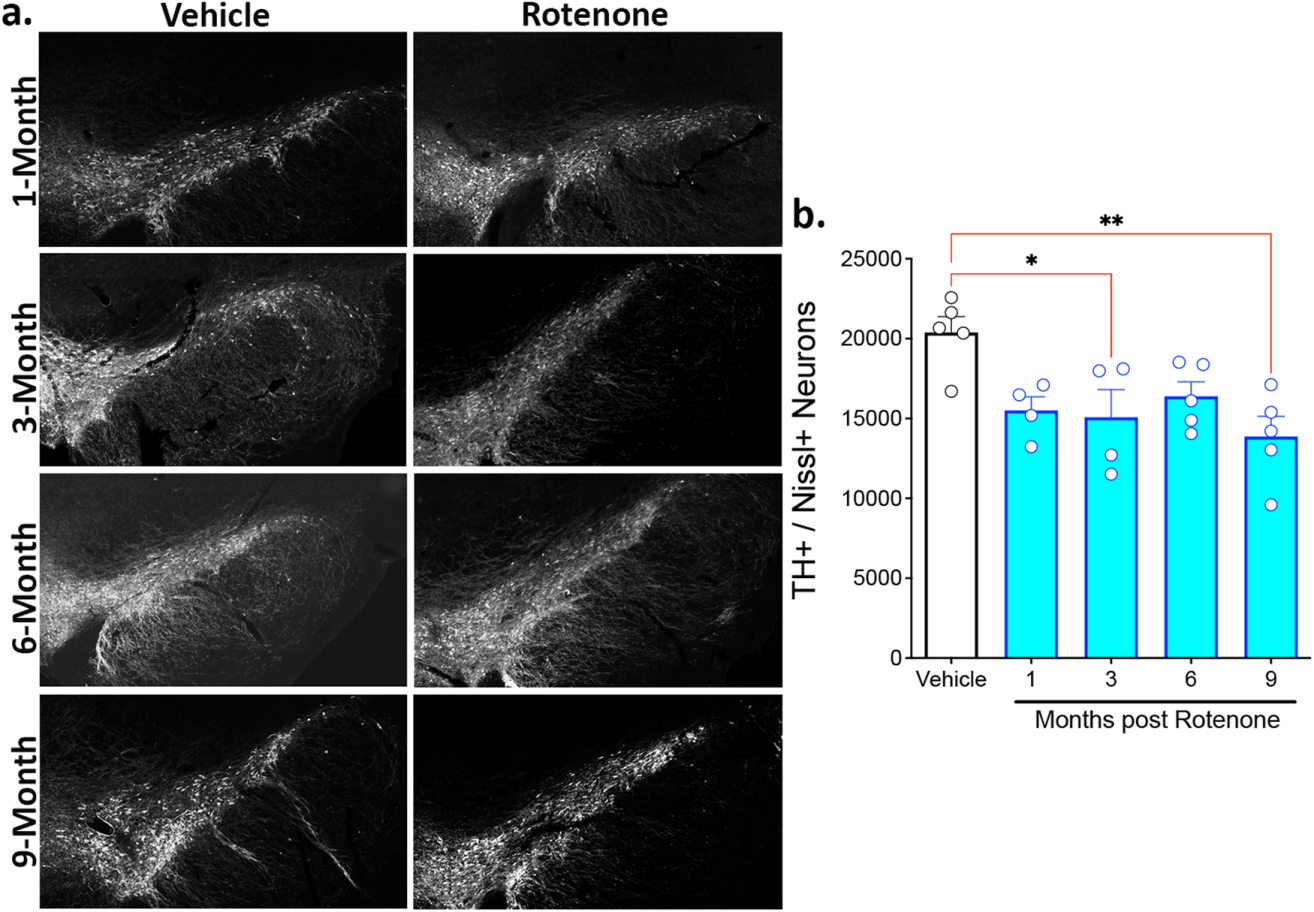
Rotenone administration triggers a loss of substantia nigra dopaminergic neurons. Representative photomicrographs of substantia nigra tyrosine hydroxylase (TH)-positive dopamine neurons from rats either 1-,3-,6-, or 9-months following a brief 5 Day exposure of either vehicle or rotenone (2.8 mg/kg, i.p.) (**a**). Stereological quantification of the surviving TH-positive dopamine neurons in the substantia nigra in all rats treated with either vehicle or rotenone (**b**). Each data symbol represents an individual brain. Graphs are expressed as mean ± SEM. Data analyzed using one-way ANOVA and Tukey multiple comparison test (*p < 0.05, **p < 0.01; *n* = 4–6 per group).

Rats were euthanized 1-, 3-, 6-, or 9-months following a brief 5-day exposure to rotenone. Using an established semi-automated stereological method^16^, the number of TH-positive and Neurotrace (fluorescent Nissl)-positive neurons in the substantia nigra were quantified. A transient exposure to rotenone resulted in loss of substantia nigra dopaminergic neuron cell bodies (*F*_4,18_ = 4.834; *P* < 0.009, one-way ANOVA). At 1-month post-rotenone, there was a non-significant (*P* < 0.06) trend toward a decrease in the number of dopamine neurons in the substantia nigra. At 3-months, the loss in dopaminergic neurons (24%) was statistically significant (*P* < 0.05) and by 9 months, there was a 32% loss of dopaminergic neurons in the SNc (*P* < 0.005, Tukey’s post-hoc comparison), Fig. 2. It is important to note, that the loss of TH-positive neurons paralleled the loss of Nissl-positive neurons. At the chosen time-points, a loss of TH immunostaining did not proceed a loss of Nissl. Although the rotenone-induced neurodegeneration was not progressive, the loss of DA neurons in the substantia nigra preceded the onset of motor symptoms and there was well-established neuropathology (loss of substantial nigra neurons and accumulation of α-synuclein puncta) by the time overt clinical abnormalities were present.

At the level of the striatum, a brief exposure of rotenone resulted in an early (1 month) reduction in striatal TH (*F*_4,19_ = 4.377; *p* < 0.05, one-way ANOVA), dopamine transporter (DAT; *F*_4,19_ = 7.403; *p* < 0.001, one-way ANOVA) and vesicular monoamine transporter 2 (VMAT2; *F*_4,19_ = 4.707; *p* < 0.03, one-way ANOVA), as assessed by fluorescence intensity. The reduction in striatal DAT-positive dopamine terminals persisted at 3- and 6-months, in comparison to controls (*p* < 0.05), but despite the persistent significant reduction in the number of dopaminergic neuronal cell bodies, striatal DAT-immunoreactivity returned to basal levels by 9-months (Supplementary Fig. 1). Similar effects were seen with TH and VMAT2 (not shown). Thus, after at least 6 months of degenerative changes in nigrostriatal nerve terminals, there may be terminal sprouting at 9 months post-rotenone.

### Delayed α-synuclein accumulation following transient rotenone exposure

**Fig. 3:**
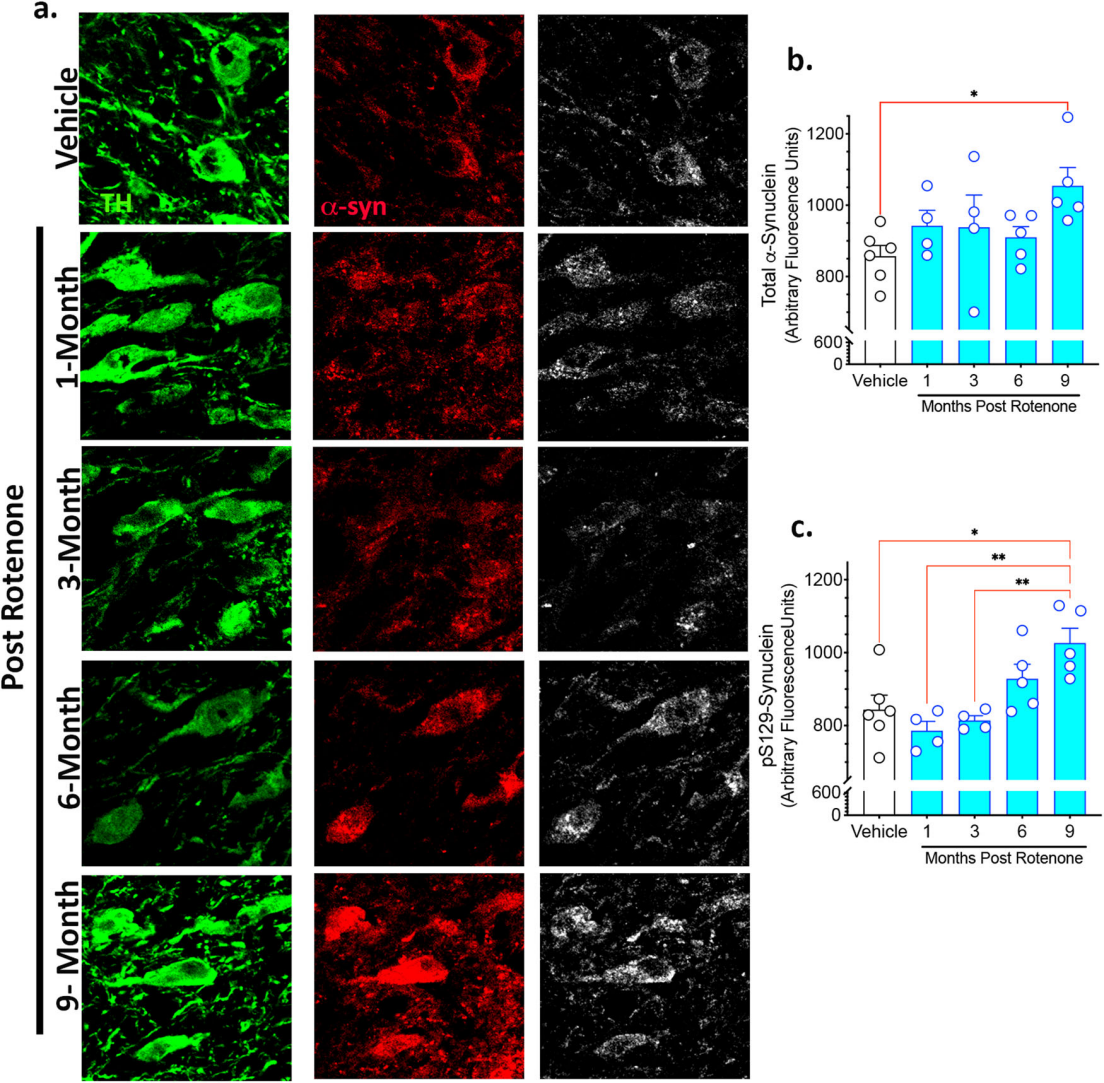
A brief rotenone exposure triggers progressive accumulation of endogenous α-synuclein in substantia nigra dopaminergic neurons. Rats were treated with vehicle or rotenone for 5 consecutive days and euthanized either 1-, 3-, 6-, or 9-months following the final injection. Endogenous α-synuclein levels were evaluated by quantitative confocal microscopy in surviving dopaminergic neurons in the substantia nigra. **a** Representative photomicrographs showing endogenous total α-synuclein (red), tyrosine hydroxylase (green; marker for dopaminergic neurons) and pSer129 α-synuclein (white). Immunofluorescence signal for total α-synuclein (**b**) or pS129- α-synuclein (**c**) was quantified in (3–4) sections for each rat. Each data symbol represents mean cellular values from an individual brain. Graphs are expressed as mean ± SEM. Data analyzed using one-way ANOVA and Tukey multiple comparison test (*p < 0.05, **p < 0.01 vs. vehicle; *n* = 4–6 per group).

Lewy bodies and Lewy neurites are a major neuropathological hallmark of PD and consist largely of the protein α-synuclein. The immunofluorescent intensity of total and phosphorylated Serine 129 (pSer129) α-synuclein was assessed in substantia nigra dopaminergic neurons in rats treated with rotenone (Fig. 3). Rotenone caused accumulation of endogenous α-synuclein that was significantly higher than vehicle-treated rats 9 months after the rotenone exposure (*F*_4,19_ = 2.436; *P* < 0.05, one-way ANOVA). In parallel, rotenone caused a significant increase in pSer129 α-synuclein in substantia nigra dopaminergic neurons (*F*_4,19_ = 6.996; *P* < 0.001, one-way ANOVA). This accumulation of pSer129 α-synuclein was delayed, becoming significant 9 months post-rotenone (*P* < 0.01, Tukey’s post-hoc comparison) (Fig. 3). All dopaminergic neurons positive for pSer129 α-synuclein were also positive for total α-synuclein.

**Fig. 4:**
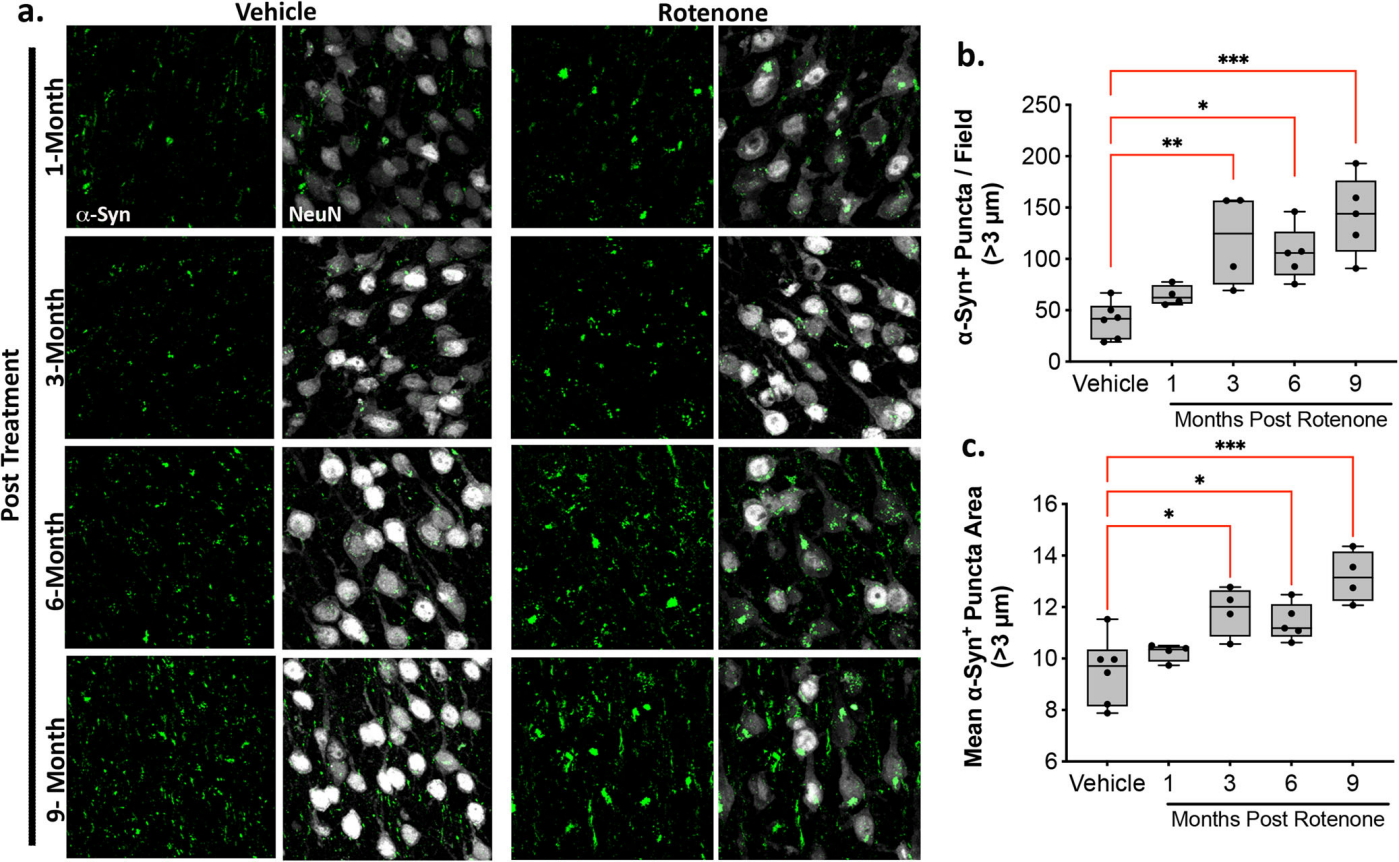
A brief rotenone exposure triggers progressive accumulation of endogenous α-synuclein in the cerebral cortex. Endogenous total α-synuclein levels were evaluated in the cortex of rats that were treated with vehicle or rotenone for 5 consecutive days and euthanized either 1-, 3-, 6-, or 9-months following the final injection. **a** Representative photomicrographs of endogenous total α-synuclein (green) punctae in NeuN-positive neuronal cell bodies (white). **b** Quantification of the number of total α-synuclein-immunoreactive punctae in NeuN positive cell bodies >3 um. **c** Quantification of the average size of each total α-synuclein-immunoreactive punctae >3 um. Each data symbol represents the mean from an individual animal. The center line of each interquartile box represents the median, the upper and lower lines of the box represent the upper and the lower quartiles, respectively. The lower and upper whiskers represent the minimum and maximum data point. Data analyzed using one-way ANOVA and Tukey multiple comparisons tests (*p < 0.05, **p < 0.01, ***p < 0.001 vs. vehicle; *n* = 4–6 per group).

**Fig. 5:**
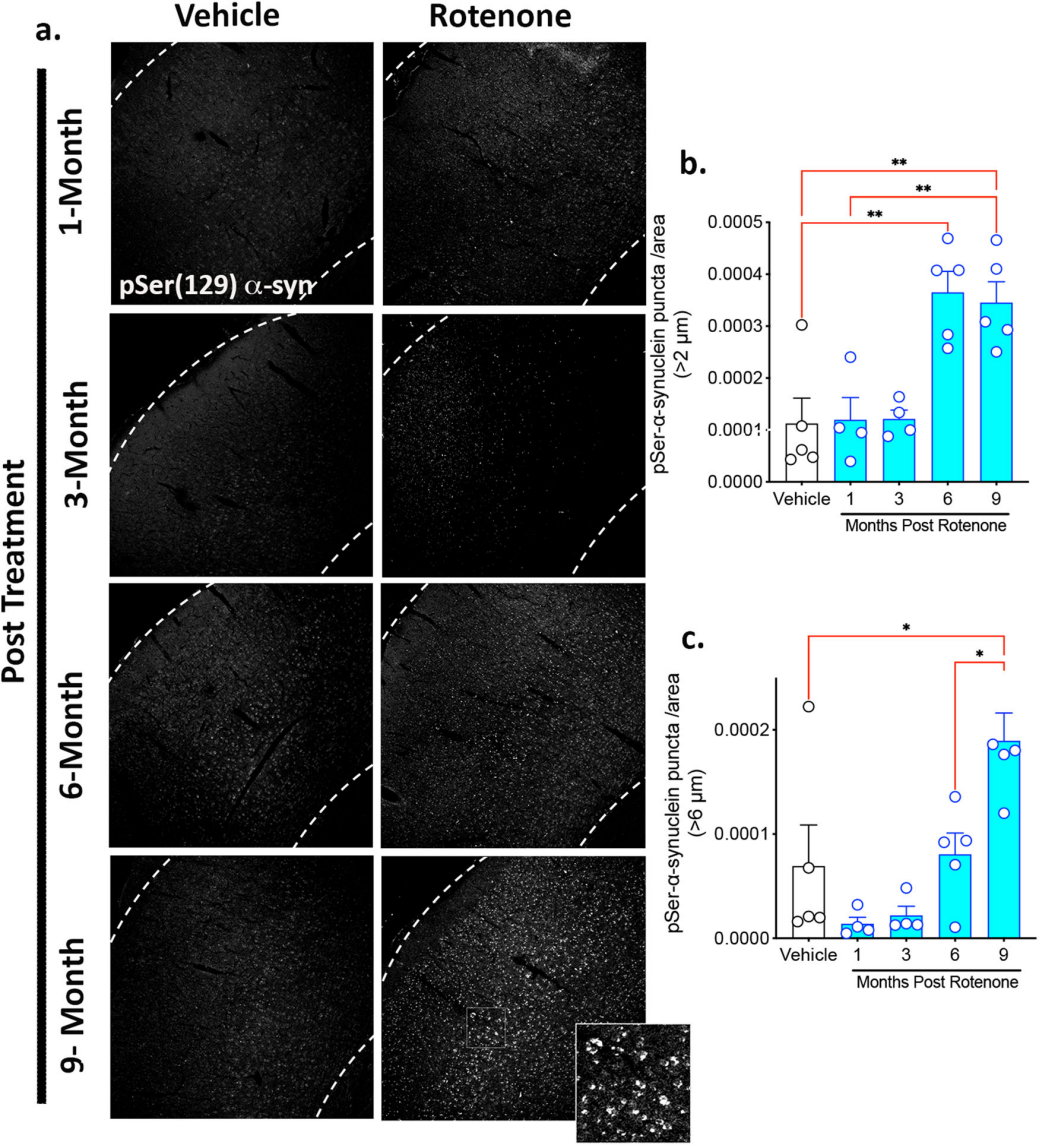
A brief rotenone exposure triggers progressive accumulation of endogenous pSer129 α-synuclein in the cerebral cortex. Endogenous α-synuclein levels were evaluated in the cortex of rats that were treated with vehicle or rotenone for 5 consecutive days and euthanized either 1-, 3-, 6-, or 9-months following the final injection. **a** Representative photomicrographs of endogenous pSer129 α-synuclein (white). Quantification of the density of pS129-immunoreactive puncta of (**b**) >2 um and (**c**) >6 um. Each data symbol represents the mean from an individual animal. Graphs are expressed as group mean ± SEM. Data analyzed using one-way ANOVA and Tukey multiple comparisons tests (*p < 0.05, **p < 0.01 vs. vehicle; *n* = 4–6 per group).

The immunofluorescent intensity of total and phosphorylated Serine 129 (pSer129) α-synuclein was assessed in the cortex of rats treated with rotenone (Figs. 4–5). Rotenone caused a progressive accumulation of endogenous total α-synuclein punctae in the cortex after the rotenone exposure (*F*_4,19_ = 2.777; *P* < 0.0001, one-way ANOVA). We assessed all α-synuclein immunoreactive punctae larger than 3 µm in diameter (Fig. 4). We found a significant accumulation of endogenous α-synuclein at 3-, 6-, and 9-months post rotenone treatment (*p* < 0.01, *p* < 0.05, *p* < 0.0001, respectively, Tukey multiple comparison test). The average size of each punctae also gradually increased in the cortex (*F*_4,18_ = 10.24; *P* < 0.0002, one-way ANOVA) reaching statistical significance at 3-, 6-, and 9-months post rotenone treatment (*p* < 0.05, *p* < 0.05, *p* < 0.0001, respectively, Tukey multiple comparison test). Consistent with cortical accumulation of neuronal pSer129-α-synuclein in individuals with PD, rats exposed to rotenone had a higher burden of pSer129-α-synuclein in the cortex compared to vehicle, especially in the middle cortical layers (Fig. 5). We assessed all pSer129-α-synuclein immunoreactive punctae larger than 2 µm in diameter as well as, separately, “larger” punctae (>6 µm diameter) and found delayed increases in both – (*F*_4,18_ = 10.51; *p* < 0.0001, one-way ANOVA) and (*F*_4,18_ = 7.488; *p* < 0.001, one-way ANOVA), respectively. Interestingly, while accumulation of pSer129-α-synuclein punctae >2 µm was significant by 6 months post-rotenone (*P* < 0.01, Tukey’s post-hoc comparison), it was only after 9 months that the increase in larger punctae (>6 µm) became significant relative to vehicle (*P* < 0.01, Tukey’s post-hoc comparison). This may indicate that individual pSer129-α-synuclein puncta, once established, enlarge over time.

### Progressive neuroinflammation in the substantia nigra following a brief rotenone exposure

**Fig. 6:**
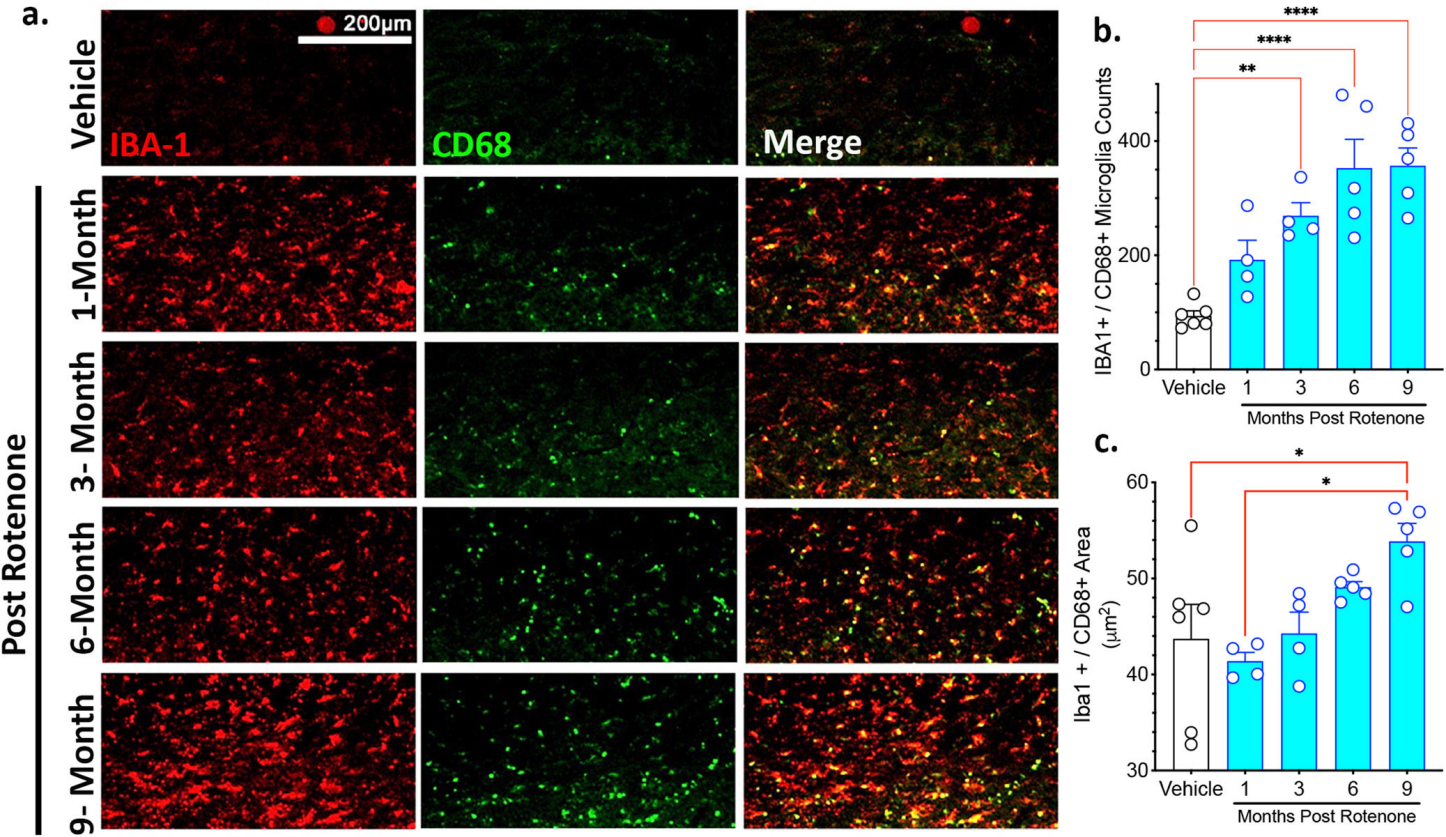
A brief rotenone exposure triggers progressive neuroinflammatory response in the substantia nigra . Rats were treated with vehicle or rotenone for 5 consecutive days and euthanized either 1-, 3-, 6-, or 9-months following the final injection. **a** Neuroinflammation was assessed in the substantia nigra using Iba-1 (red) as a marker of microglia and CD68 (green) as a marker of phagocytic and reactive microglia. **b** The number of phagocytic and reactive microglia was assessed by quantifying the number of Iba-1-positive microglia that were also CD68-positive. **c** An additional measure of phagocytic and reactive microglia was assessed by reported the size (mm^2^) of each Iba1-positive and CD68-positive microglia. Each data symbol represents an individual brain. Graphs are expressed as mean ± SEM. Data analyzed using one-way ANOVA with Tukey multiple comparisons test (*p < 0.05, **p < 0.01, ***p < 0.001, ****p < 0.0001; *n* = 4–6 per group).

Neuroinflammation and increases in reactive microglia are well established features of PD^17,18^. We used Iba-1 as a marker of all microglia and CD68, a lysosomal protein that is up-regulated in reactive microglia, to assess the number and activational state of microglia over time (Fig. 6). Rotenone caused a progressive, time-dependent increase in the number of reactive microglia in the substantia nigra compared to vehicle treated rats (*F*_4,19_ = 13.94; *p* < 0.0001, one-way ANOVA). The number of Iba-1 positive and CD68-positive microglia was significantly increased 3-months following rotenone as compared to vehicle (*P* < 0.01, Tukey’s post-hoc comparison). The number of Iba-1 and CD68-positive reactive microglia continued to increase and eventually peaked at 6-months (*P* < 0.0001 Tukey’s post-hoc comparison) and remained stable at 9-months post-rotenone treatment (*P* < 0.0001 Tukey’s post-hoc comparison). Consistent with previously reported changes in morphology as microglia become reactive^19,20^, the size of the microglial soma became larger as they became more reactive (*F*_4,19_ = 4.260; *p* < 0.05, one-way ANOVA) and peaked at 9-months (*P* < 0.05 Tukey’s post-hoc comparison) following exposure to rotenone (Fig. 5).

## Discussion

Capturing the complexities of a neurodegenerative condition like PD in a single animal model is extremely challenging and justifies the need for additional, time-linked and clinically relevant parkinsonian models. An animal model that produces neurodegeneration, progressive α-synuclein pathology and neuroinflammation coupled with delayed motor disturbances would be a valuable preclinical tool. Such a model would allow for the testing of therapeutic interventions at clinically relevant timepoints and, ultimately, may improve predictive value of therapeutic testing at the preclinical stage. In this context, we sought to refine the preclinical rotenone rat model.

Current understanding of disease pathogenesis strongly suggests that idiopathic PD may result from an interaction between genetic risk variants and environmental factors^12^. Epidemiological studies link exposure to environmental toxicants – like rotenone, paraquat and trichloroethylene – to increased risk of PD, even though the exposures may have occurred decades before the emergence of symptoms^9,10,21,22^. It is known that rodents exposed to these toxicants develop some of the hallmark features of the disease, such as accumulation of endogenous α-synuclein, nigral neuroinflammation, and DA neuron neurodegeneration^7,8,14,23^. Although these animal models have provided insight into the molecular mechanisms that render dopaminergic neurons preferentially vulnerable, they are relatively acute – causing dopaminergic degeneration in a matter of days to weeks. In contrast, our revised rotenone model results in neuropathological changes that present months after a transient (5 day) rotenone exposure and continue to increase for at least 9 months. At 1-month post-rotenone exposed rats displayed a loss of DA neurons in the substantia nigra that remained stable up to 9-months post-rotenone. Although, this loss is not progressive, a unique component of this model is the progressive gait disturbance that emerged without further manipulation or treatment 3-months after rotenone exposure, which continued to worsen for the duration of the study (Fig. 1).

Individuals with PD typically receive a diagnosis once they have developed motor symptoms, at which point there is already significant loss of substantia nigra dopamine neurons, early α-synuclein accumulation, and ongoing neuroinflammation. In this context, it is noteworthy that coincident with the appearance of motor deficits in rotenone rats (a milestone that might be considered roughly equivalent to appearance of motor symptoms in people with PD) there was already nigral cell loss, ongoing inflammation, and an upward trend of α-synuclein levels . As such, the appearance of motor symptoms at about 90 days post-rotenone provides an interesting timepoint at which to initiate potential disease-modifying therapeutic strategies. In other words, by the time motor dysfunction is detected, the ‘disease process’ is well underway – and without intervention, the parkinsonian syndrome will continue to progress behaviorally and pathologically. This raises the important question of whether therapeutic intervention at this clinically relevant behavioral timepoint can stabilize motor function and slow or stop pathogenic processes and pathological outcomes. Ongoing studies will address this issue.

Accumulation of endogenous α-synuclein within the substantia nigra and cortex is consistent with that observed in post-mortem PD tissue. We reported previously that 5 days of rotenone treatment causes an acute increase of α-synuclein in nigrostriatal neurons when assayed on day 6^14^. In contrast, here we first assayed α-synuclein at 1 month, approximately 25 days after cessation of rotenone treatment, and found levels comparable with those found in vehicle treated rats. This may suggest that, in the short term, there is effective α-synuclein proteostasis. Alternatively, it is possible that the nigrostriatal neurons that had an early rise in α-synuclein may have died by the time α-synuclein was assessed in the current study. By 9 months, however, there was a significant increase in both total and pSer129-α-synuclein in substantia nigra dopaminergic neurons and cortical neurons compared to vehicle treated rats. Aggregates positive for pSer129-α-synuclein have been observed in the cortex of individuals with PD both histologically^24^ and in homogenates of post-mortem PD tissue^25–27^. Therefore, we measured pSer129-α-synuclein in this region. Cortical pSer129-α-synuclein had a laminar distribution that was most prominent in middle layers of frontal cortex. Aggregates 2 µm or larger accumulated significantly by 6 months and larger aggregates (>6 µm) were seen at 9 months, suggesting progressive α-synuclein dyshomeostasis. Thus, in both the substantia nigra and cortex, a transient, temporally-remote, exposure to an environmental toxicant can cause a delayed and progressive endogenous synucleinopathy.

Neuroinflammation has long been implicated in PD pathogenesis, in part supported by the presence of activated microglia that reside in abundance within the substantia nigra of PD patients^28^. It is also well established that rotenone can cause an inflammatory response in vivo^29,30^. Moreover, rotenone can activate microglia via proinflammatory signaling pathways^31–33^. Rats that received a brief (5 day) treatment of rotenone had a delayed and gradual increase in the number of CD68-positive reactive microglia. At 3-months post-rotenone exposure, when motor dysfunction was emerging, the number of CD68-positive reactive microglia was significantly increased and, as more time elapsed, the number and size of the reactive microglia increased and then plateaued between 6–9 months post-rotenone.

There is strong epidemiological evidence that exposure to environmental toxicants, such as rotenone, paraquat or trichloroethylene increases the risk of developing PD, but the fact that such exposures may occur years before emergence of symptoms has been somewhat perplexing. In our model, a brief exposure to rotenone clearly sets in motion an inflammatory neurodegenerative process and a delayed synucleinopathy that only manifests behaviorally with motor dysfunction 3 months after exposure. Thus, our results provide a possible explanation as well as ‘biological plausibility’ for the epidemiological findings of a ‘latent’ period between exposure and clinical diagnosis.

By necessity, much of our understanding of PD has come from studying patients well into the course of disease. In part, current drug discovery and therapeutic development is limited by the preclinical models in use, which generally recapitulate pathologies associated with late-stage PD and have unproven predictive value. Additionally, in these models, it is common practice for therapeutic interventions to be administered prior to or concomitant with neurotoxic insults (e.g., toxicants or genetic manipulations) that model underlying disease factors. In contrast, the model described here has the potential for therapeutic intervention at more clinically relevant points in disease evolution, such as when motor symptoms first emerge and when there is already ongoing neurodegeneration. Given current clinical efforts to diagnose PD earlier, before motor symptoms arise, it is worth noting that our proposed model also allows for testing during the prodromal latent period between exposure and development of motor impairment.

There remains much to learn about this new model, including an understanding of how this rotenone dosing paradigm compares in female rats^29^ or disease-relevant genetically-modified animals. It is also not known whether the delayed accumulation of α-synuclein in the cortex is associated with cognitive deficits in rats exposed to rotenone. If so, it raises the clinically relevant possibility of testing whether therapeutic intervention initiated when motor symptoms are first detectable (i.e., at ‘diagnosis’) can prevent or delay onset of non-motor symptoms like cognitive dysfunction. It is also not clear if rats exposed to only 5- doses of rotenone gradually develop gastrointestinal deficits, similar to rats repeatedly dosed rotenone. As such, this model holds great potential with many outstanding questions, and the full utility of this animal model remains to be determined.

## Methods

### Animals and supplies

All animal procedures were approved by the Institutional Animal Care and Use Committee (IACUC) at the University of Pittsburgh and are in accordance with guidelines delineated by the National Institutes of Health in the *Guide for the Care and Use of Laboratory Animals*. Previous work from our group showed that female rats require higher doses of rotenone to produce an equivalent degree of nigrostriatal degeneration and, even at these higher doses, females showed less inflammation and less accumulation of α-synuclein^29^. As such, there was substantial uncertainty about the dosing and duration of rotenone treatment in females in relation to this new treatment regime. For this reason, we used only male rats in this initial feasibility study. Lewis rats, aged 7–9 months, were obtained from Envigo (Indianapolis, IN). Upon arrival, two weeks prior to the start of the study, rats were single-housed and handled daily to prevent stress upon study commencement. Conventional diet and water were available to rats *ad libitum*, and animals were maintained under standard temperature-controlled conditions with a 12-hour light-dark cycle. All chemicals, unless stated otherwise, were purchased from Sigma-Aldrich (St. Louis, MO, USA).

### Rotenone treatment

Rotenone was dissolved in dimethylsulfoxide (DMSO) and diluted in medium-chain triglyceride, Miglyol 812N (Sasol North America, Inc) to reach a final concentration of 2.8 mg/mL rotenone in 98% Miglyol 812N, 2% DMSO. The final working concentration of rotenone was made fresh every 2 days and stored at room temperature shielded from light. After 2 weeks of behavioral training (described below), rats received once-daily i.p. injections of rotenone (2.8 mg/kg i.p.) or vehicle (98% Miglyol 812N, 2% DMSO) for 5 consecutive days.

### Postural instability testing

All rats were trained for 2 weeks prior to rotenone administration to establish baseline performance on the Postural Instability Test (PIT), as previously described^13,15^. For this testing, rats were held by the examiner perpendicularly to a sandpaper board marked with 0.5 cm intervals. One forelimb was allowed to contact a sandpaper board marked and the rat’s nose was placed at the “0” cm mark. Each rat was encouraged to moved slowly and steadily along the surface until a compensatory forelimb movement was triggered. The distance at which the forelimb movement was completed was recorded. Rats were randomly assigned a code number and all blinded assessments were performed in triplicate and then averaged. Initially, rats underwent daily PIT assessments; however, the frequency gradually tapered to a single weekly assessment around 8 weeks following final rotenone exposure. Weekly PIT assessment continued until each rat reached their pre-determined endpoint.

### Immunohistochemistry

Rats were deeply anesthetized using pentobarbital (50 mg/kg) and were perfused using 0.1 M ice-cold PBS and perfused using 4% paraformaldehyde, pH 7.4. Brains were post-fixed for 24 h and then placed in 30% sucrose at 4 °C. Sucrose saturated brains were sectioned on a frozen sliding microtome at 35 µm thickness and stored in cryoprotectant at −20 °C until use. Immunofluorescent histological analyses were carried out using the following primary antibodies against α-synuclein (1:1000, BD 610787), Tyrosine hydroxylase (TH; 1:2000, Millipore MAB1542), NeuN (1:1000, Abcam ab104224), phosphorylated Serine129 α-synuclein (1:1000, Millipore AB51253), Iba-1 (1:1000, Abcam AB5076) and CD68 (1:1000, Biorad MCA3416A); and secondary antibodies, used at a concentration of 1:500, Cy3-conjugated anti-mouse (Jackson Immuno), Alexa Fluor 488 (Molecular Probes), and Cy5-conjugated anti-rabbit (Jackson Immuno). All Images were acquired using an Olympus FV-1000 confocal microscope utilizing constant laser and detector settings that were optimized to avoid pixel saturation.

### Striatal terminal fluorescence intensity

Dopaminergic terminal density was assessed in rat brain sections (35 µm) using a 1/6 sampling fraction, encompassing the entire striatum (approximately 8–10 sections per animal). Free floating striatal tissue sections were incubated in a primary antibody against TH (1:2000 mouse anti-TH; Millipore MAB318) for 36 h at 4 °C. The sections were then washed in PBS for 3 × 10 min and incubated at room temperature for 1 hr with donkey anti-mouse secondary antibody (1:500, LI-COR, IRdye 800CW). Sections were then washed and mounted on glass slides using gelvatol mounting media. Sections were imaged using a LI-COR infrared scanner and analyzed using the LI-COR Odyssey Analysis software, regions of interest were drawn in the striatum, excluding the nucleus accumbens, and mean fluorescence intensity was measured.

### Unbiased stereology

Dopaminergic neuron survival was assessed in blinded fashion using a well-established semi-automated protocol^14,16,34^ and 1/6 sampling fraction, encompassing the entire substantia nigra (approximately 8–10 sections per animal). Briefly, substantia nigra tissue sections were stained for TH (sheep anti-TH 1:2000 Millipore MAB1542) and counterstained with DAPI and Nissl NeuroTrace Dye (640; Life Technologies) then imaged using an Olympus BX61VS automated slide-scanner microscopy system with a 20× objective (NA 0.75) to obtain extended focal images with height of 26 µm and 1 µm step size. Images were stitched together using Olympus vs-asw 2.9.2 software. The system is equipped with a Marzhauser Wetzlar automated slide scanning stage, an X-Cite 120 LED boost light source and an Allied Vision Pike F-505C VC50 camera. Stereological analysis was achieved using a semi-automated system rather than the optical fractionator method. 20X images were imported into Nikon NIS-Elements Advanced Research software (Version 4.5, Nikon, Melville, NY). An ROI was drawn around the substantia nigra and each image underwent a background subtraction and thresholding for each individual fluorescent channel. The number of TH-positive neurons that overlapped with Nissl (Neurotrace-positive) quantified. Results are reported as the number of TH-positive cell bodies (whole neurons) within the substantia nigra.

Similar methodology was used to quantify the number of reactive microglia within the substantia nigra. Briefly, 3–4 midbrain sections from a single hemisphere were captured using an Olympus BX61VS automated slide-scanner microscopy system with a 20× objective (NA 0.75). Images were stitched together using Olympus vs-asw 2.9.2 software. These images were imported into Nikon NIS-Elements Advanced Research software (Version 4.5, Nikon, Melville, NY). A region of interest was drawn around the substantia nigra, excluding the pars reticulata and the thresholding tool was applied to label Iba1-positive microglia and CD68-positive microglia. The number of Iba1 microglia co-labeled with CD68 were quantified and considered reactive microglia.

### Statistical analyses

During the experimental design stage, a-priori power analyses were conducted using G*power software (Heinrich-Heine-University Düsseldorf) to determine sample size required to achieve adequate power, with a 95% power at α = 0.05. For statistical comparisons between vehicle and rotenone groups, a one-way analysis of variance (one-way ANOVA) was conducted. If overall significant P-value and F-statistic was achieved, additional Tukey post-hoc tests were used for multiple pairwise comparisons. All data are expressed as mean values ± standard error of the mean (SEM). Statistical significance between treatment groups is represented in each figure as *p < 0.05, **p < 0.01, ***p < 0.001, ****p < 0.0001. Statistical analyses were carried out using GraphPad Prism software (Version 9).

### Supplementary information

Supplementary Material

## Data Availability

The data that supports the findings in this manuscript are available by contacting the corresponding authors.
