# Supplementary material for: Transient exposure to rotenone causes degeneration and progressive parkinsonian motor deficits, neuroinflammation, and synucleinopathy
Source: NPJ Parkinsons Dis. 2023 Aug 11;9:121. doi: 10.1038/s41531-023-00561-6 (PMC10421849; doi:10.1038/s41531-023-00561-6)
Supplement: Supplementary file 1 — Supplementary Material [file 41531_2023_561_MOESM1_ESM.pdf]

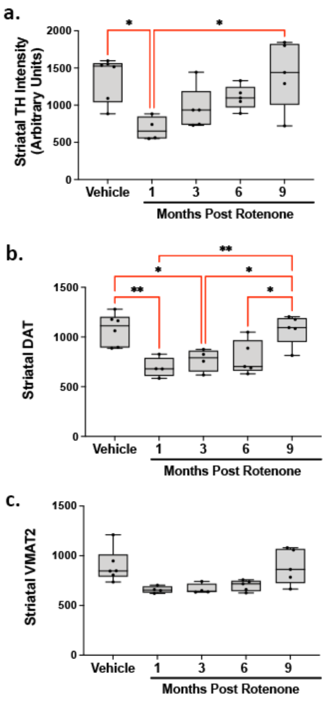

Supplementary Figure 1. Time course of striatal DAT levels after rotenone. Dopaminergic terminal density was assessed in rat brain sections using a 1/6 sampling fraction, encompassing the entire striatum (approximately 8-10 sections per animal). Sections were imaged using a LI-COR infrared scanner and analyzed using the LI-COR Odyssey Analysis software, regions of interest were drawn in the striatum, excluding the nucleus accumbens, and mean fluorescence intensity was measured. Each data symbol represents the mean from an individual animal. The center line of each interquartile box represents the median, the upper and lower lines of the box represent the upper and the lower quartiles, respectively. The lower and upper whiskers represent the minimum and maximum data point. Statistical analysis by one-way ANOVA with Tukey multiple comparisons test (*$p<0.05$, **$p<0.01$; n= 4-6 per group). Similar results were obtained for TH and VMAT2 fluorescence intensities (not shown).
